# Beyond Rensch’s Rule: Prevalent Female-Biased Size Dimorphism and Its Allometric Scaling in Cassidinae Beetles

**DOI:** 10.3390/insects17020208

**Published:** 2026-02-16

**Authors:** Jialong Wang, Yuru Yang, Chaokun Yang, Chengqing Liao, Jiasheng Xu, Qingyun Guo, Xiaohua Dai

**Affiliations:** 1 Leafminer Group, School of Life Sciences, Gannan Normal University, Ganzhou 341000, China; 15319641926@163.com (J.W.); 18434283054@163.com (Y.Y.); yang1764847081@163.com (C.Y.); liaochengqing@gnnu.edu.cn (C.L.); gnnxjs@163.com (J.X.); guoqingyun@gnnu.edu.cn (Q.G.); 2 National Navel Orange Engineering Research Center, Ganzhou 341000, China

**Keywords:** sexual size dimorphism (SSD), body size, leaf beetles, Rensch’s rule, Nanling Mountains

## Abstract

Body size is a key trait influencing life history and ecological adaptation, and sexual size dimorphism (SSD) reflects divergent selective pressures acting on males and females. In morphologically conserved insect groups such as Cassidinae leaf beetles, the external similarity between sexes often impedes accurate dimorphism assessment. To address this, we conducted a systematic morphometric study of ten Cassidinae species from the Nanling Mountains—the largest east–west mountain system in southern China—where we definitively assigned sex via genital dissection. We measured body weight, body length, body width, length–width ratio, and corresponding wing traits. Across all species, SSD was consistently female biased, with statistically significant but subtle differences in most traits; body weight exhibited the greatest relative disparity. While this pattern aligns with the fecundity advantage hypothesis, direct fecundity data were not collected. Crucially, interspecific allometric analyses revealed that the scaling of male and female body sizes was statistically indistinguishable from that of isometry, providing no significant support for Rensch’s rule in this female-biased system. Our findings offer foundational insights into SSD evolution in cryptically dimorphic, herbivorous beetles and highlight the need for phylogenetically informed studies across broader geographic and taxonomic scales.

## Simple Summary

In the animal world, many species exhibit differences in body size between males and females—a phenomenon known as sexual size dimorphism (SSD). A well-known macroevolutionary pattern, Rensch’s rule, predicts how SSD scales with body size depending on which sex is larger. This study investigated SSD in tortoise and hispine beetles (Cassidinae), a group of leaf beetles from the Nanling Mountains of southern China, where males and females are extremely similar. Using genital dissection for reliable sex identification, we measured body and wing dimensions across ten species. Females are consistently larger than males—a pattern often associated with fecundity selection in insects, although direct reproductive data are lacking. Importantly, the allometric scaling of SSD showed no significant support for Rensch’s rule, suggesting that body size evolution in these morphologically conserved beetles may follow distinct evolutionary pathways.

## 1. Introduction

Body size is a key indicator reflecting an organism’s environmental adaptability and evolutionary history [1,2,3]. As the most fundamental and conspicuous functional trait of organisms, body size influences individual physiology and behavior; affects intra- and interspecific interactions; and thereby shapes the structure and dynamics of populations, communities, and ecosystems [1,2,4,5,6,7]. Global changes are accelerating the evolution of organismal body size [5,6,8]. Previous ecological studies on animal body size have focused primarily on vertebrates, particularly mammals, but now increasingly emphasize invertebrates, especially insects [9,10]. The research scale has expanded from the individual and population levels to the community and ecosystem levels, enhancing our understanding of the ecological role of body size [7]. Body size encompasses both magnitude and shape. Since body size magnitude is easier to measure, research on its ecological implications has been more thorough than studies on shape [8]. Under the long-term combined influence of genetic and environmental factors, the body size of organisms may exhibit divergent patterns [11], which often follow certain ecological rules, such as Bergmann’s rule and the converse Bergmann rule [2,8,12], James’ rule and Atkinson’s rule [8,13], Allen’s rule and the converse Allen rule [2], Rensch’s rule and the converse Rensch rule [14,15,16,17], and Cope’s rule [18]. Many ecological rules related to body size essentially reflect the relationship between body size and energy (temperature, heat, and evapotranspiration). Since individual metabolic rates can be derived from body size and temperature [19], ecological metabolic theory can be used to analyze the formation mechanisms of body size patterns [13,20,21].

Sexual size dimorphism (SSD), a systematic difference in body size between males and females, represents a fundamental aspect of phenotypic diversity with significant implications for life history, ecology, and evolution [22]. Within the hyperdiverse beetle family Chrysomelidae, SSD has complex and varied manifestations. In several subfamilies, especially Cryptocephalina, pronounced sexual size dimorphism is often accompanied by extreme functional and morphological specializations. These adaptations, such as enlarged male mandibles for combat or modified tarsi for grasping females during copulation, are typically interpreted as outcomes of intense sexual selection [23,24,25,26,27]. In contrast, SSD within the subfamily Cassidinae has received comparatively limited research attention and has seldom been examined within a broad comparative framework [28]. Most Cassidinae species lack conspicuous sex-specific weaponry or ornaments [28], suggesting that their SSD, where present, may be driven primarily by genetic drift or natural selection rather than by direct intrasexual competition or mate choice [29]. Previous studies reporting body length and width in Cassidinae beetles have rarely distinguished between males and females [30].

A central macroevolutionary pattern in SSD is Rensch’s rule. In taxa where males are relatively large, SSD increases with body size, whereas in female-larger taxa, SSD decreases as body size increases [14,15,31]. Although originally proposed for vertebrates, the rule has since been examined across diverse animal groups. However, empirical support in arthropods remains inconsistent and notably scarce relative to their extraordinary species diversity [32,33]. Importantly, most insect studies have adopted two contrasting approaches: broad comparisons across distantly related orders or families [32,33] or fine-scale intraspecific analyses of single species [34]. Both overlook a critical intermediate scale—the comparison among closely related species within a diverse subfamily—where shared ecology and phylogeny can reveal lineage-specific allometric patterns that broader or narrower comparisons may miss.

The leaf-beetle subfamily Cassidinae, with over 6400 species globally and approximately 500 recorded in China, is the second largest in Chrysomelidae [28,35]. Although female-biased SSD has been noted in some species [28], no systematic multi-species assessment has examined whether this pattern is consistent across tribes or how it scales with body size. Moreover, Cassidinae beetles generally lack conspicuous external sexual traits, making reliable sex identification difficult without dissection. This cryptic dimorphism can introduce bias in SSD studies that rely solely on external morphology.

To address these knowledge gaps, we conducted the first systematic investigation of SSD and Rensch’s rule across the Cassidinae subfamily. Using ten species representing multiple tribes from the Nanling Mountains in China and employing genital dissection for definitive sex identification, this study aims to (1) establish a robust, comparative quantitative baseline of SSD within this morphologically conserved group and (2) explicitly test whether the allometric scaling of SSD in the female-larger Cassidinae species conforms to the prediction of Rensch’s rule. Our findings provide essential descriptive data to frame future investigations into the evolutionary drivers (e.g., natural selection vs. genetic drift) of body-size dimorphism in lineages lacking overt sexual selection.

## 2. Materials and Methods

### 2.1. Study Area

The Nanling Mountains form the largest east–west mountain system in southern China and serve as a key biogeographic transition between the Central Chinese and South Chinese floristic regions. Characterized by a subtropical monsoon climate and extensive evergreen broad-leaved forests, the region harbors exceptional plant diversity—including numerous host families of Cassidinae—and high insect species richness and endemism [36,37,38,39,40]. This ecological heterogeneity makes the Nanling Mountains an ideal setting for studying sexual size dimorphism in a representative assemblage of leaf beetles.

Field surveys focused on the eastern Nanling Mountains, primarily around Ganzhou (Jiangxi Province). An additional site, Dinghu Mountain (Guangdong Province), lies just outside the formal range but within its biogeographic sphere; sufficient samples of one Callispini species were collected there for inclusion in our analyses.

### 2.2. Study Species

On the basis of preliminary field surveys, one or two representative species were selected from each of the eight common tribes within the subfamily Cassidinae (Table 1), covering all four subfamilies sensu Chen, 1986 [30]. The chosen species are relatively large in size and abundant in number within their respective tribes. To cover the full range of size variations, an additional species was included for each of the two tribes—Aspidomorphini and Basiprionotini—which consist of particularly large beetles. No distinct diagnostic characteristics were observed between males and females in the 10 species of Cassidinae beetles examined. All the studied species are medium- to large-sized Cassidinae, with adult body lengths ranging from approximately 4 to 13 mm. To minimize potential biases due to geographic and interannual variations, all the samples were collected from the same region—the eastern Nanling Mountains—during a single continuous 12-month period (August 2024–July 2025). Moreover, for each species, males and females were obtained from the same collection events and processed simultaneously, ensuring temporal and procedural consistency.

Sampling sites were selected to prioritize habitats with minimal to moderate human disturbance and well-preserved vegetation. Host plants of Cassidinae beetles were systematically searched along field trials, and leaves were inspected for signs of insect activity, such as larval mines or feeding marks. Upon detection, the entire plant and neighboring plants were examined. Adults, larvae or pupae were collected in the field together with their host plant material and immediately placed into sealed zip-lock bags to maintain humidity and viability during transport [41,42]. In *Notosacantha sauteri*, adults are rarely encountered in the field; therefore, final-instar larvae or pupae were collected along with fresh host branches and leaves and reared to adulthood under laboratory conditions. All other species were collected directly as adults.

Adult specimens were morphologically identified with reference to the reference manuals [30,43,44]. Difficult taxa were verified by Cassidinae specialists: Dr. Charles L. Staines (Smithsonian Environmental Research Center, Edgewater, MD, USA) and Dr. Lukáš Sekerka (National Museum, Prague, Czech Republic). All the insect samples were deposited in the Nanling Insect Collection, Gannan Normal University, Ganzhou, China (GNNU).

### 2.3. Body Size Measurement

Body weight, body length, body width, wing length and wing width were measured as morphometric proxies for overall body size. Adult beetles were kept alive under ambient laboratory conditions and weighed as live fresh mass using a Sartorius BSA124S precision balance (±0.1 mg, Mettler Toledo International Inc., Zurich, Switzerland) shortly after removal from the zip-lock bags. No fasting, hydration standardization, or desiccation protocols were applied, as individuals were subsequently preserved in absolute ethanol (Xilong Scientific Co., Ltd., Shantou, China) for downstream DNA extraction and genetic analyses.

**Figure 1:**
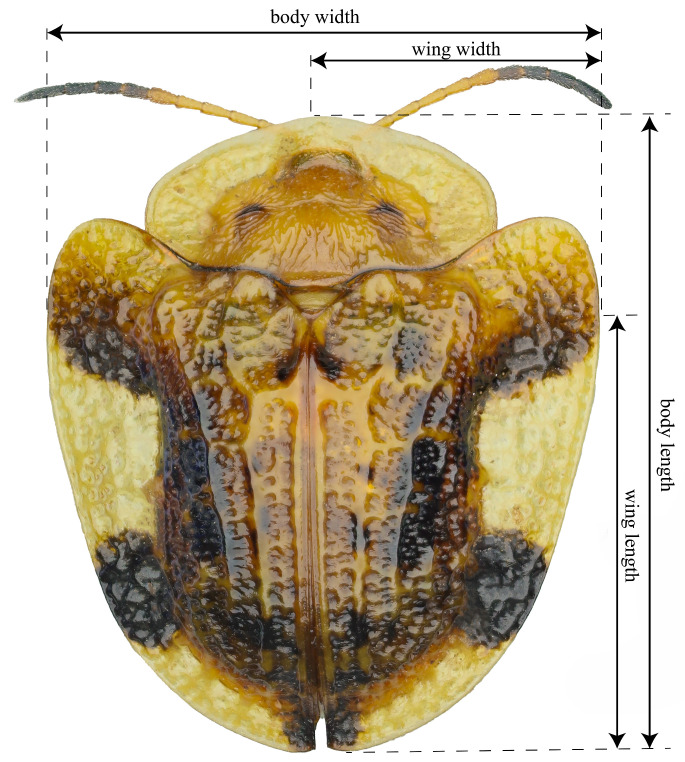
Standardized measurement protocols for body size and wing size parameters.

The measurement standards for linear size parameters are illustrated in Figure 1. Body length was measured from the midpoint of the anterior margin of the frons (between the eyes, extending to the anterior margin of the pronotum in Cassidinae beetles) to the posterior apex of the elytra along the dorsal midline. Body width was measured at the maximum transverse diameter across the pronotum and elytra, including the lateral margins of the elytra, but excluding spine length in the case of Hispini beetles. Wing length was measured along the dorsal midline from the apex of the scutellum (the junction of the two wings) to the posterior end of the elytral suture. Wing width was measured as the maximum width of the elytra perpendicular to the suture, including the epipleural margin but excluding the lateral spine length in the case of Hispini beetles. Photographs were taken with a Canon EOS 70D camera (Canon Inc., Tokyo, Japan) with an MP-E 65 mm f/2.8 1–5× macro lens (Canon Inc., Tokyo, Japan). The captured images were analyzed via Digimizer 6.5.0 software to measure body length and width. Sex identification was performed via an Olympus SZX16 stereomicroscope (Olympus Corporation, Tokyo, Japan) and dissection needles. All the data are provided in File S1 (body size) and File S2 (wing size).

### 2.4. Dissection and Sex Identification

**Figure 2:**
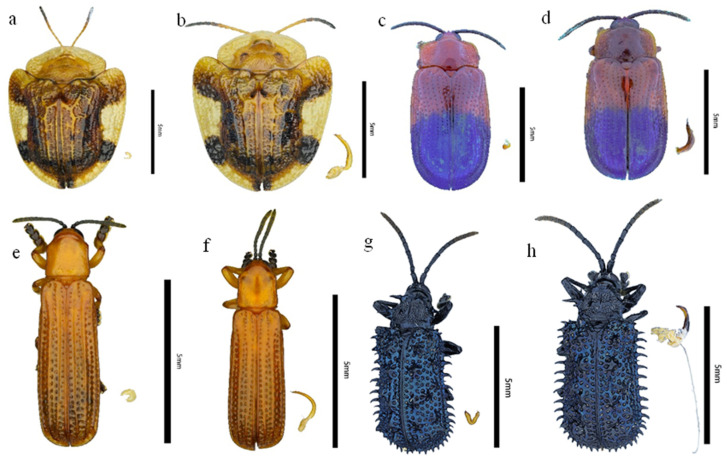
Some insects of the subfamily Cassidinae and their genitalia. (**a**): Female *Laccoptera quadrimaculata* with its spermatheca; (**b**): Male *Laccoptera quadrimaculata* with its aedeagus; (**c**): Female *Callispa dimidiatipennis* with its spermatheca; (**d**): Male *Callispa dimidiatipennis* with its aedeagus; (**e**): Female *Downesia tarsata* with its spermatheca; (**f**): Male *Downesia tarsata* with its aedeagus; (**g**): Female *Monohispa tuberculata* with its spermatheca; (**h**): Male *Monohispa tuberculata* with its aedeagus. Scale bar = 5 mm.

All morphometric measurements were completed prior to dissection to ensure data integrity and avoid any alteration of body dimensions. The samples were then immersed in absolute ethanol. Using homemade dissecting needles and VETUS ST-14 forceps (Shanghai Vetus Tool Co., Ltd., Shanghai, China), the last three abdominal sternites were removed to expose the internal cavity. The complete reproductive system was then extracted, and sex was definitively determined by identifying the presence of an aedeagus (male) or a spermatheca (female) under a microscope. After dissection, each sample was preserved in absolute ethanol in an individual glass vial. The genitalia of Cassidinae were photographed separately via an Olympus SZX16 stereomicroscope for future use. Figure 2 shows representative Cassidinae adults and their corresponding genitalia.

### 2.5. Data Analysis

The dataset was compiled by matching each sample’s predissection measurements with its postdissection sex determination. For each Cassidinae species, sex-related differences in body weight, body length, body width, the body length–width ratio, wing length, wing width and the wing length-width ratio were quantified via arithmetic means of each trait per sex. To assess the statistical significance of these differences, independent two-sample *t* tests (assuming equal variances unless Levene’s test indicated otherwise) were performed. The use of means—rather than medians—was appropriate given the approximately normal distribution of log-transformed morphometric traits and the parametric nature of our analytical framework. To examine whether allometric scaling between sexes conforms to Rensch’s rule, standardized major axis (SMA) regression analyses were performed via the Smatr 4.5.2 R package [45]. Following approaches used in many previous SSD studies, SMA regressions for each trait were fitted, with log-transformed female values used as the independent variable and log-transformed male values used as the dependent variable [14,46]. Under the observed female-biased SSD, conformity with Rensch’s rule was indicated by a slope significantly greater than 1. Conversely, a slope significantly less than 1 indicates that the degree of female-biased SSD increases with overall body size, which corresponds to Rensch’s rule [14,15,33,46]. For each regression, the overall model significance (*p* value), coefficient of determination (*R*^2^), and 95% confidence interval of the slope were evaluated. A permutation-based hypothesis test was conducted to determine whether each slope differed significantly from 1 (i.e., isometry). Support for Rensch’s rule was inferred if the confidence interval excluded 1, the associated *p* value was less than 0.05, and the slope estimate was greater than 1. Sexual size dimorphism (SSD) was quantified as the relative difference between sexes and was calculated as [(♀ mean − ♂ mean)/♀ mean] × 100%. Positive values denote female-biased SSD.

All the statistical analyses and visualizations were performed via R version 4.5.2 [47] within the RStudio IDE 2023.12.1.402 [48]. The R packages dplyr 1.1.4, tidyr 1.3.1, and readxl 1.4.3 were used for data input, manipulation, and output [49,50,51]. The R packages ggplot2 3.5.1 and ggpubr 0.6.0 were employed to generate graphical outputs [52,53].

## 3. Results

### 3.1. Sexual Size Dimorphism in Cassidinae Species

#### 3.1.1. *Aspidomorpha sanctaecrucis*

**Figure 3:**
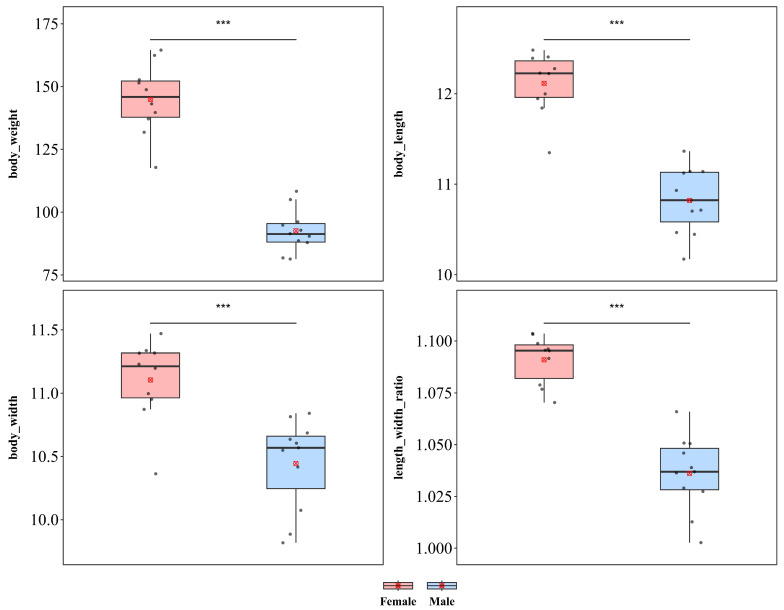
Sexual size dimorphism in *Aspidomorpha sanctaecrucis.* Boxplots showing the distributions of body length, body width, body weight, and length–width ratio in female and male adults. The box represents the interquartile range, the horizontal line inside indicates the median, the whiskers extend to 1.5× the interquartile range, and the circles denote potential outliers. Significance markers are based on independent two-sample *t* tests: *** *p* < 0.001.

*A*. *sanctaecrucis* presented significant sexual dimorphism in terms of body length, body width, and body weight (Figure 3). Compared with males, females were significantly greater in weight (♀ *n* = 10, $\bar{x}$ = 144.880 mg, *sd* = 14.210; ♂ *n* = 11, $\bar{x}$ = 92.582 mg, *sd* = 8.419; *t* = 10.380), body length (♀ $\bar{x}$ = 12.114 mm, *sd* = 0.342; ♂ $\bar{x}$ = 10.820 mm, *sd* = 0.363; *t* = 8.387), and body width (♀ $\bar{x}$ = 11.105 mm, *sd* = 0.323; ♂ $\bar{x}$ = 10.445 mm, *sd* = 0.358; *t* = 4.413), with all differences reaching extremely significant levels (*p* < 0.001), indicating a pattern of female-biased sexual size dimorphism. Additionally, a significant difference in the length–width ratio was detected between the sexes (♀ $\bar{x}$ = 1.091, *sd* = 0.012; ♂ $\bar{x}$ = 1.036, *sd* = 0.018; *t* = 8.230, *p* < 0.001), indicating that the increase in female body size was not isometric scaling and that female body morphology was more elongated than that of males.

*A. sanctaecrucis* also exhibited significant sexual dimorphism in wing length, wing width, and the wing length–width ratio (File S3). Compared with males, females presented significantly greater values of both wing length (♀ $\bar{x}$ = 7.953 mm, *sd* = 0.423; ♂ $\bar{x}$ = 6.741 mm, *sd* = 0.351; *t* = 7.173, *p* < 0.001) and wing width (♀ $\bar{x}$ = 5.638 mm, *sd* = 0.308; ♂ $\bar{x}$ = 5.242 mm, *sd* = 0.249; *t* = 43.262), with all differences reaching significant levels (*p* < 0.01). Additionally, a significant difference in the wing length–width ratio (♀ $\bar{x}$ = 1.413, *sd* = 0.088; ♂ $\bar{x}$ = 1.287, *sd* = 0.064; *t* = 3.776; *p* < 0.01) was detected between the sexes, indicating that female wings were more elongated than male wings were.

#### 3.1.2. *Laccoptera quadrimaculata*

**Figure 4:**
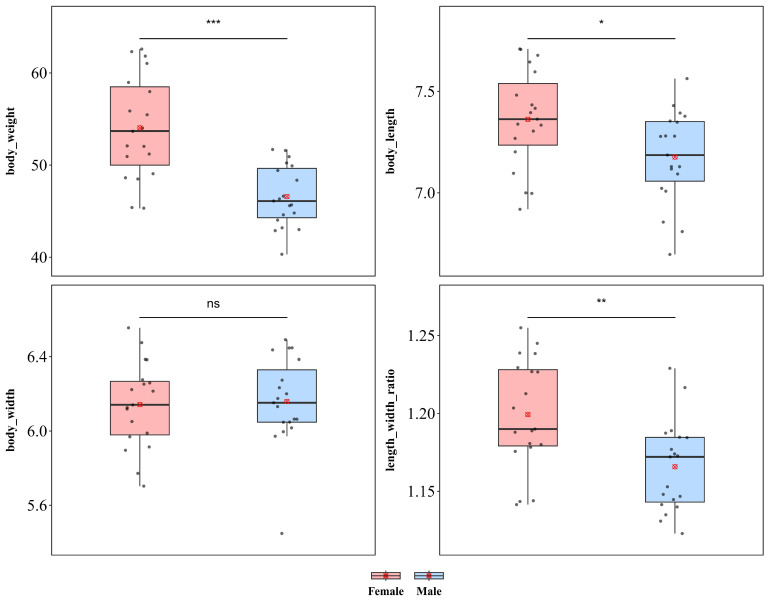
Sexual size dimorphism in *Laccoptera quadrimaculata.* *** *p* < 0.001, ** *p* < 0.01, * *p* < 0.05, ns indicates not significant.

*L. quadrimaculata* showed significant sexual dimorphism in body length and body weight but not in body width (Figure 4). Compared with males, females were significantly greater in weight (♀ *n* = 19, $\bar{x}$ = 54.05 mg, *sd* = 5.566; ♂ *n* = 19, $\bar{x}$ = 46.59 mg, *sd* = 3.321; *t* = 5.016, *p* < 0.001) and body length (♀ $\bar{x}$ = 7.362 mm, *sd* = 0.244; ♂ $\bar{x}$ = 7.176 mm, *sd* = 0.227; *t* = 2.435, *p* < 0.05). However, there was no significant difference in body width between the sexes (♀ $\bar{x}$ = 6.142 mm, *sd* = 0.231; ♂ $\bar{x}$ = 6.159 mm, *sd* = 0.242; *t* = −0.222, *p* = 0.825). Additionally, a significant difference in the length–width ratio was detected between the sexes (♀ $\bar{x}$ = 1.199, *sd* = 0.035; ♂ $\bar{x}$ = 1.166, *sd* = 0.029; *t* = 3.198, *p* < 0.01), indicating that the increase in female body size was not isometric scaling and that female body morphology was more elongated than that of males.

*L. quadrimaculata* also exhibited significant sexual dimorphism in terms of wing length and the LWR, while no significant difference in wing width was detected (File S3). Compared with male individuals, female individuals presented significantly greater wing length (♀ $\bar{x}$ = 6.274 mm, *sd* = 0.303; ♂ $\bar{x}$ = 5.940 mm, *sd* = 0.350; *t* = 3.138, *p* < 0.01). In contrast, no significant difference was detected in body width (♀ $\bar{x}$ = 3.647 mm, *sd* = 0.206; ♂ $\bar{x}$ = 3.578 mm, *sd* = 0.239; *t* = 0.956, *p* = 0.345). Additionally, a significant difference in the wing length–width ratio (♀ $\bar{x}$ = 1.723 mm, *sd* = 0.079; ♂ $\bar{x}$ = 1.663 mm, *sd* = 0.072; *t* = 2.444; *p* < 0.05) was observed between the sexes, indicating that female wings were more elongated than male wings were.

#### 3.1.3. *Basiprionota bisignata*

**Figure 5:**
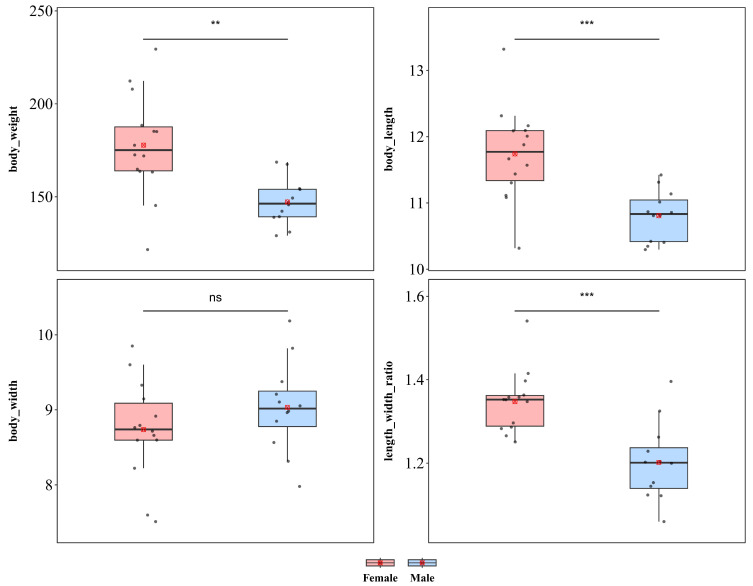
Sexual size dimorphism in *Basiprionota bisignat*. *** *p* < 0.001, ** *p* < 0.01, ns indicates not significant.

*B. bisignata* showed significant sexual dimorphism in body length and body weight but not in body width (Figure 5). Compared with males, females were significantly greater in weight (♀ *n* = 14, $\bar{x}$ = 177.80 mg, *sd* = 27.503; ♂ *n* = 12, $\bar{x}$ = 147.24 mg, *sd* = 12.498; *t* = 3.732, *p* < 0.01) and body length (♀ $\bar{x}$ = 11.74 mm, *sd* = 0.708; ♂ $\bar{x}$ = 10.809 mm, 0.379; *t* = 4.266, *p* < 0.001). However, there was no significant difference in body width between the sexes (♀ $\bar{x}$ = 8.735 mm, *sd* = 0.660; ♂ $\bar{x}$ = 9.033 mm, *sd* = 0.602; *t* = −1.195, *p* > 0.05). Additionally, a significant difference in the length–width ratio was detected between the sexes (♀ $\bar{x}$ = 1.347, *sd* = 0.074; ♂ $\bar{x}$ = 1.202, *sd* = 0.093; *t* = 4.440, *p* < 0.001), indicating that the increase in female body size was not isometric scaling and that female body morphology was more elongated than that of males.

*B. bisignata* also exhibited significant sexual dimorphism in terms of wing length and the wing length–width ratio, whereas no significant difference was detected in wing width (File S3). Compared with male individuals, female individuals presented significantly greater wing length (♀ $\bar{x}$ = 8.802 mm, *sd* = 0.482; ♂ $\bar{x}$ = 7.904 mm, *sd* = 0.487; *t* = 4.715; *p* < 0.001). In contrast, no significant difference was detected in body width (♀ $\bar{x}$ = 4.963 mm, *sd* = 0.314; ♂ $\bar{x}$ = 4.816 mm, *sd* = 0.315; *t* = 1.185; *p* = 0.248). Additionally, a significant difference in the wing length–width ratio (♀ $\bar{x}$ = 1.777 mm, *sd* = 0.109; ♂ $\bar{x}$ = 1.645 mm, *sd* = 0.119; *t* = 2.947; *p* < 0.01) was observed between the sexes, indicating that female wings were more elongated than male wings were.

#### 3.1.4. *Basiprionota chinensis*

**Figure 6:**
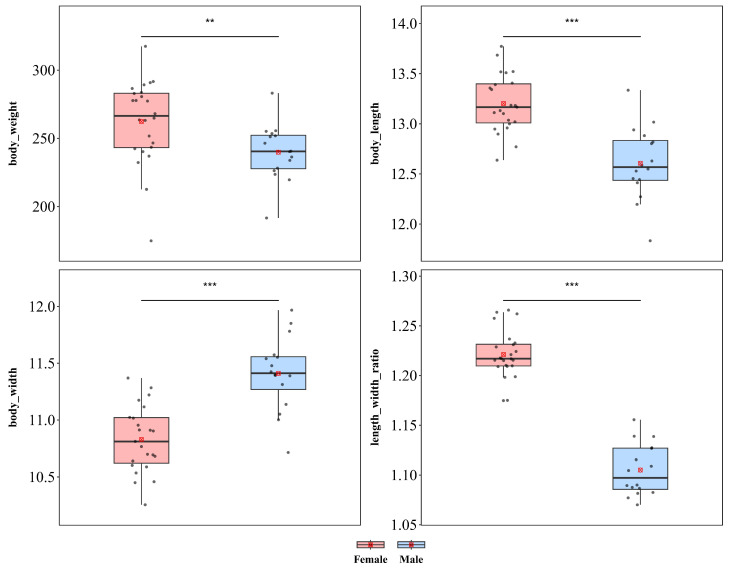
Sexual size dimorphism in *Basiprionota chinensis*. *** *p* < 0.001, ** *p* < 0.01.

*B. chinensis* showed significant sexual dimorphism in terms of body length, body weight, and body width (Figure 6). Females were significantly greater in weight (♀ *n* = 24, $\bar{x}$ = 262.38 mg, *sd* = 30.399; ♂ *n* = 16, $\bar{x}$ = 239.84 mg, *sd* = 20.389; *t* = 2.596, *p* < 0.01) and body length (♀ $\bar{x}$ = 13.309 mm, *sd* = 0.593; ♂ $\bar{x}$ = 12.606 mm, *sd* = 0.360; *t* = 4.234, *p* < 0.001). However, the trend was reversed for body width, with males being significantly wider than females (♀ $\bar{x}$ = 10.900 mm, *sd* = 0.449; ♂ $\bar{x}$ = 11.411 mm, *sd* = 0.324; *t* = −3.913, *p* < 0.001). Additionally, a significant difference in the length–width ratio was detected between the sexes (♀ $\bar{x}$ = 1.221, *sd* = 0.024; ♂ $\bar{x}$ = 1.105, *sd* = 0.026; *t* = 14.476, *p* < 0.001), indicating that the increase in female body size was not isometric scaling and that female body morphology was more elongated than that of males.

*B. chinensis* also exhibited significant sexual dimorphism in wing length, wing width, and the wing length–width ratio (File S3). Compared with male individuals, female individuals presented significantly greater wing length (♀ $\bar{x}$ = 10.214 mm, *sd* = 0.411; ♂ $\bar{x}$ = 9.501 mm, *sd* = 0.536; *t* = 4.515; *p* < 0.001), whereas no significant difference was observed in body width (♀ $\bar{x}$ = 5.883 mm, *sd* = 0.206; ♂ $\bar{x}$ = 6.162 mm, *sd* = 0.807; *t* = −1.350; *p* = 0.195). Additionally, females presented a significantly greater wing length–width ratio (♀ $\bar{x}$ = 1.737 mm, *sd* = 0.068; ♂ $\bar{x}$ = 1.558 mm, *sd* = 0.151; *t* = 4.463; *p* < 0.001) than males did, indicating that female wings were more elongated than male wings were.

#### 3.1.5. *Callispa dimidiatipennis*

**Figure 7:**
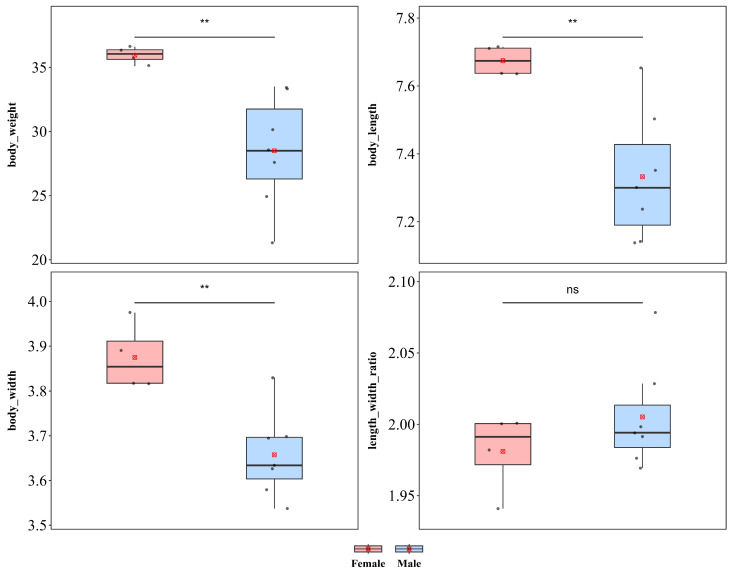
Sexual size dimorphism in *Callispa dimidiatipennis*. ** *p* < 0.01, ns indicates not significant.

*C. dimidiatipennis* showed significant sexual dimorphism in body length, body width, and body weight (Figure 7). Compared with males, females were significantly greater in weight (♀ *n* = 4, $\bar{x}$ = 35.95 mg, *sd* = 0.656; ♂ *n* = 7, $\bar{x}$ = 28.50 mg, *sd* = 4.367; *t* = 4.428), body length (♀ $\bar{x}$ = 7.674 mm, *sd* = 0.044; ♂ $\bar{x}$ = 7.333 mm, *sd* = 0.190; *t* = 4.553), and body width (♀ $\bar{x}$ = 3.875 mm, *sd* = 0.075; ♂ $\bar{x}$ = 3.657 mm, *sd* = 0.096; *t* = 3.885), with all differences reaching extremely significant levels (*p* < 0.01), indicating a pattern of female-biased sexual size dimorphism. In contrast, the body length–width ratio did not differ between sexes (♀ $\bar{x}$ = 1.981, *sd* = 0.028; ♂ $\bar{x}$ = 2.005, *sd* = 0.037; *p* = 0.293), indicating that the larger body size of females might result from isometric scaling without alterations in body shape.

*C. dimidiatipennis* showed no significant sexual dimorphism in terms of wing length, wing width, or the wing length–width ratio (File S3). Compared with male individuals, females presented no significant differences in either wing length (♀ $\bar{x}$ = 6.048 mm, *sd* = 0.226; ♂ $\bar{x}$ = 5.922 mm, *sd* = 0.253; *t* = 0.782; *p* = 0.454) or wing width (♀ $\bar{x}$ = 2.182 mm, *sd* = 0.154; ♂ $\bar{x}$ = 2.095 mm, *sd* = 0.059; *t* = 1.081; *p* = 0.348). Furthermore, no significant difference in the wing length–width ratio (♀ $\bar{x}$ = 2.779 mm, *sd* = 0.165; ♂ $\bar{x}$ = 2.826 mm, *sd* = 0.074; *t* = −0.670; *p* = 0.520) was detected between the sexes.

#### 3.1.6. *Thlaspida biramosa*

**Figure 8:**
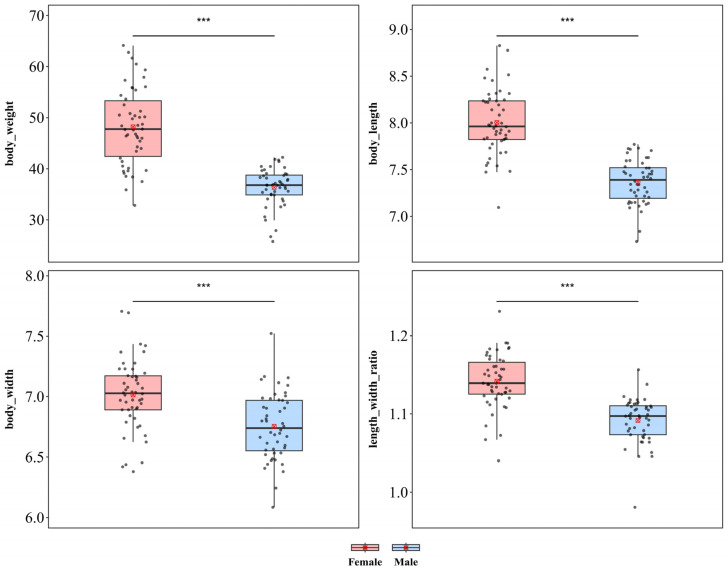
Sexual size dimorphism in *Thlaspida biramosa*. *** *p* < 0.001.

*T. biramosa* showed significant sexual dimorphism in terms of body length, body width, and body weight (Figure 8). Compared with males, females were significantly greater in weight (♀ *n* = 55, $\bar{x}$ = 48.19 mg, *sd* = 7.412; ♂ *n* = 48, $\bar{x}$ = 36.33 mg, *sd* = 3.737; *t* = 9.984), body length (♀ $\bar{x}$ = 8.005 mm, *sd* = 0.345; ♂ $\bar{x}$ = 7.368 mm, *sd* = 0.234; *t* = 10.725), and body width (♀ $\bar{x}$ = 7.014 mm, *sd* = 0.290; ♂ $\bar{x}$ = 6.754 mm, *sd* = 0.277; *t* = 4.533), with all differences reaching extremely significant levels (*p* < 0.001), indicating a pattern of female-biased sexual size dimorphism. Additionally, a significant difference in the length–width ratio was detected between the sexes (♀ $\bar{x}$ = 1.142, *sd* = 0.035; ♂ $\bar{x}$ = 1.092, *sd* = 0.029; *t* = 7.722, *p* < 0.001), indicating that the increase in female body size was not isometric scaling and that female body morphology was more elongated than that of males.

*T. biramosa* also exhibited significant sexual dimorphism in terms of wing length and width, whereas no significant difference was detected in the wing length–width ratio (File S3). Compared with male individuals, female individuals presented significantly greater wing length (♀ $\bar{x}$ = 6.048 mm, *sd* = 0.680; ♂ $\bar{x}$ = 5.564 mm, *sd* = 0.425; *t* = 3.951; *p* < 0.001) and wing width (♀ $\bar{x}$ = 3.889 mm, *sd* = 0.198; ♂ $\bar{x}$ = 3.804 mm, *sd* = 0.192; *t* = 2.004; *p* < 0.05). Additionally, a significant difference in the wing length–width ratio (♀ $\bar{x}$ = 1.556 mm, *sd* = 0.165; ♂ $\bar{x}$ = 1.465 mm, *sd* = 0.118; *t* = 2.955; *p* < 0.01) was observed between the sexes.

#### 3.1.7. *Downesia tarsata*

**Figure 9:**
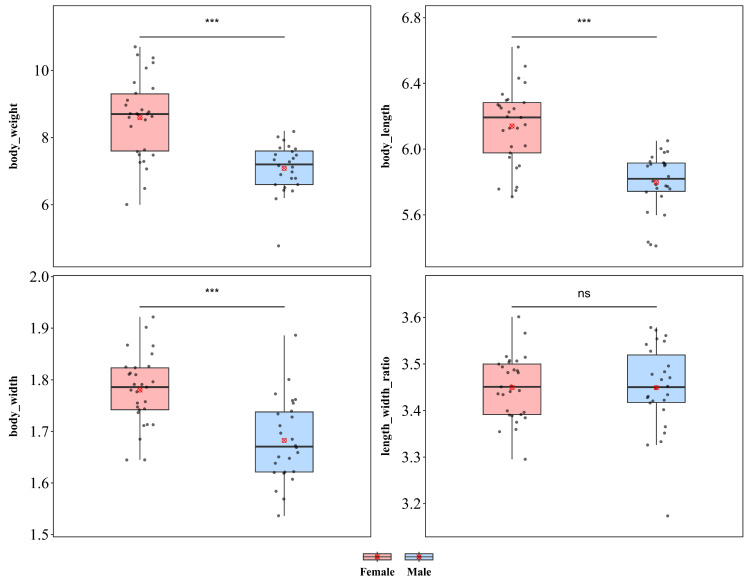
Sexual size dimorphism in *Downesia tarsata*. *** *p* < 0.001, ns indicates not significant.

*D. tarsata* showed significant sexual dimorphism in body length, body width, and body weight (Figure 9). Compared with males, females were significantly greater in weight (♀ *n* = 29, $\bar{x}$ = 8.59 mg, *sd* = 1.198; ♂ *n* = 26, $\bar{x}$ = 6.94 mg, *sd* = 0.946; *t* = 5.497), body length (♀ $\bar{x}$ = 6.140 mm, *sd* = 0.234; ♂ $\bar{x}$ = 5.798 mm, *sd* = 0.176; *t* = 6.031), and body width (♀ $\bar{x}$ = 1.780 mm, *sd* = 0.068; ♂ $\bar{x}$ = 1.683 mm, *sd* = 0.080; *t* = 4.891), with all differences reaching extremely significant levels (*p* < 0.001), indicating a pattern of female-biased sexual size dimorphism. In contrast, the body length–width ratio did not differ between sexes (♀ $\bar{x}$ = 3.450, *sd* = 0.068; ♂ $\bar{x}$ = 3.449, *sd* = 0.092; *p* = 0.011), indicating that the larger body size of females might result from isometric scaling without alterations in body shape.

*D. tarsata* showed no significant sexual dimorphism in wing length, wing width, or the wing length–width ratio (File S3). Compared with male individuals, females presented no significant differences in wing length (♀ $\bar{x}$ = 4.705 mm, *sd* = 0.198; ♂ $\bar{x}$ = 4.800 mm, *sd* = 0.191; *t* = −1.802; *p* = 0.077), wing width (♀ $\bar{x}$ = 0.916 mm, *sd* = 0.084; ♂ $\bar{x}$ = 0.951 mm, *sd* = 0.055; *t* = −1.878; *p* = 0.066), or the wing length–width ratio (♀ $\bar{x}$ = 5.199 mm, *sd* = 0.736; ♂ $\bar{x}$ = 5.059 mm, *sd* = 0.243; *t* = 0.970; *p* = 0.339). Furthermore, no significant difference in the wing length–width ratio was detected between the sexes.

#### 3.1.8. *Monohispa tuberculata*

**Figure 10:**
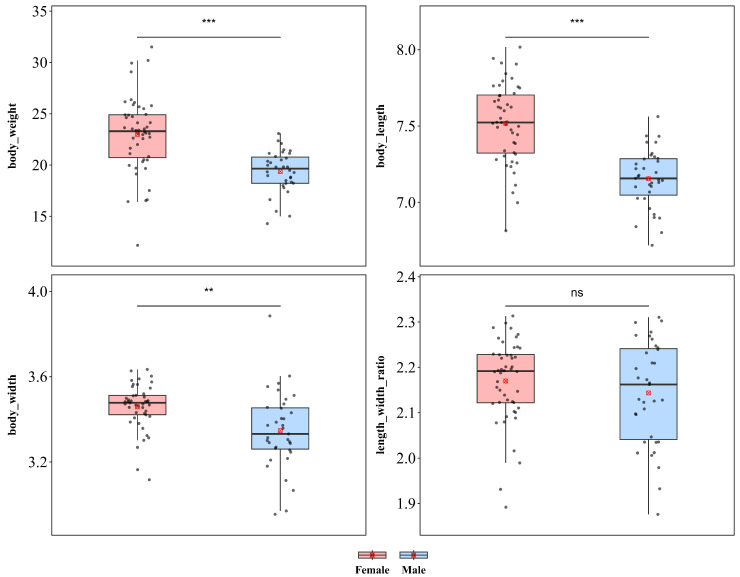
Sexual size dimorphism in *Monohispa tuberculata*. *** *p* < 0.001, ** *p* < 0.01, ns indicates not significant.

*M. tuberculata* showed significant sexual dimorphism in body length, body width, and body weight (Figure 10). Compared with males, females were significantly greater in weight (♀ *n* = 48, $\bar{x}$ = 22.99 mg, *sd* = 3.695; ♂ *n* = 35, $\bar{x}$ = 19.75 mg, sd = 3.009; *t* = 4.262), body length (♀ $\bar{x}$ = 7.518 mm, *sd* = 0.262; ♂ $\bar{x}$ = 7.155 mm, *sd* = 0.193; *t* = 6.933), and body width (♀ $\bar{x}$ = 3.469 mm, *sd* = 0.151; ♂ $\bar{x}$ = 3.346 mm, *sd* = 0.182; *t* = 3.351), with all differences reaching extremely significant levels (*p* < 0.01), indicating a pattern of female-biased sexual size dimorphism. In contrast, the body length–width ratio did not differ between the sexes (♀ $\bar{x}$ = 2.170, *sd* = 0.091; ♂ $\bar{x}$ = 2.144, *sd* = 0.114; *p* = 0.245), indicating that the larger body size in females might result from isometric scaling without alterations in body shape. *tuberculata* also exhibited significant sexual dimorphism in terms of wing length and width, whereas no significant difference was detected in the LWR (File S3). Compared with male individuals, females presented significantly greater values of both wing length (♀ $\bar{x}$ = 5.347 mm, *sd* = 0.203; ♂ $\bar{x}$ = 5.076 mm, *sd* = 0.219; *t* = 5.800; *p* < 0.001) and wing width (♀ $\bar{x}$ = 1.702 mm, *sd* = 0.104; ♂ $\bar{x}$ = 1.622 mm, *sd* = 0.103; *t* = 3.469; *p* < 0.001). Furthermore, no significant difference in the wing length–width ratio (♀ $\bar{x}$ = 3.152 mm, *sd* = 0.208; ♂ $\bar{x}$ = 3.141 mm, *sd* = 0.226; *t* = 0.223; *p* = 0.824) was detected between the sexes.

#### 3.1.9. *Leptispa longipennis*

**Figure 11:**
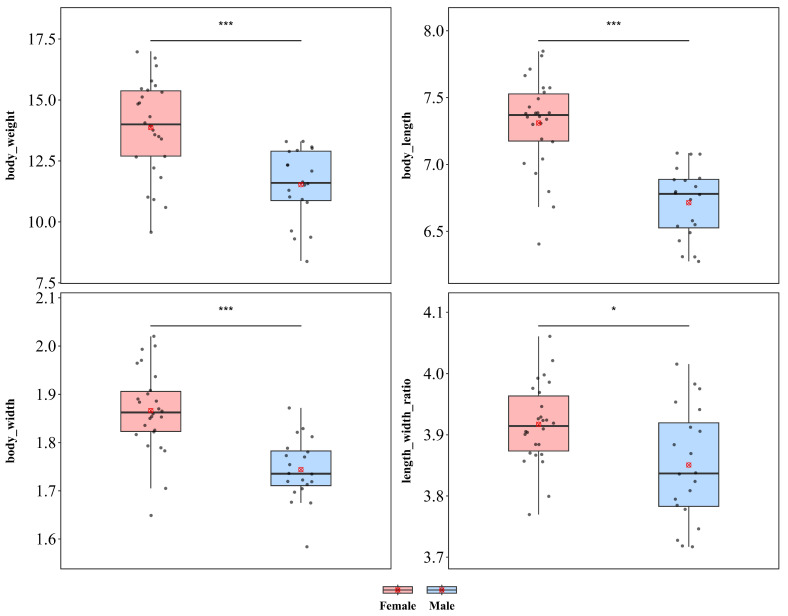
Sexual size dimorphism in *Leptispa longipennis*. *** *p* < 0.001, * *p* < 0.05.

*L. longipennis* showed significant sexual dimorphism in terms of body length, body width, and body weight (Figure 11). Compared with males, females were significantly greater in weight (♀ *n* = 26, $\bar{x}$ = 13.87 mg, *sd* = 0.066; ♂ *n* = 20, $\bar{x}$ = 11.69 mg, *sd* = 0.093; *t* = 4.436), body length (♀ $\bar{x}$ = 7.310 mm, *sd* = 0.341; ♂ $\bar{x}$ = 6.714 mm, *sd* = 0.263; *t* = 6.467), and body width (♀ $\bar{x}$ = 1.866 mm, *sd* = 0.086; ♂ $\bar{x}$ = 1.744 mm, *sd* = 0.065; *t* = 5.281), with all differences reaching extremely significant levels (*p* < 0.001), indicating a pattern of female-biased sexual size dimorphism. Additionally, a significant difference in the length–width ratio was detected between the sexes (♀ $\bar{x}$ = 3.917, *sd* = 0.066; ♂ $\bar{x}$ = 3.851, *sd* = 0.093; *t* = 2.840, *p* < 0.05), indicating that the increase in female body size was not isometric scaling and that female body morphology was more elongated than that of males.

*L. longipennis* exhibited significant sexual dimorphism in terms of wing length, whereas no significant differences were detected in wing width or the wing length–width ratio (File S3). Compared with male individuals, female individuals presented significantly greater wing length (♀ $\bar{x}$ = 5.982 mm, *sd* = 0.352; ♂ $\bar{x}$ = 5.601 mm, *sd* = 0.393; *t* = 3.461; *p* < 0.01). Additionally, no significant differences were detected in wing width (♀ $\bar{x}$ = 1.034 mm, *sd* = 0.084; ♂ $\bar{x}$ = 1.000 mm, *sd* = 0.094; *t* = 1.305; *p* = 0.199) or the wing length–width ratio (♀ $\bar{x}$ = 5.801 mm, *sd* = 0.311; ♂ $\bar{x}$ = 5.627 mm, *sd* = 0.403; *t* = 1.659; *p* = 0.104) between the sexes.

#### 3.1.10. *Notosacantha sauteri*

**Figure 12:**
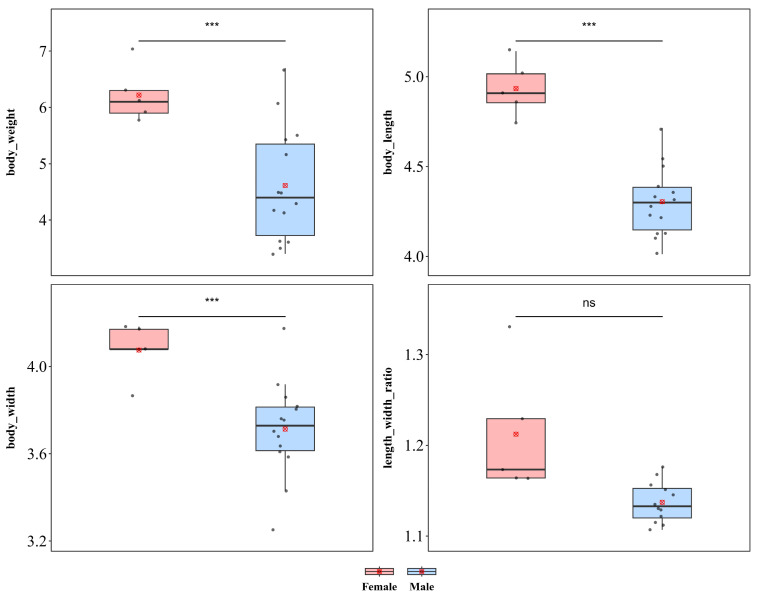
Sexual size dimorphism in *Notosacantha sauteri.* *** *p* < 0.001, ns indicates not significant.

*N. sauteri* showed significant sexual dimorphism in body length, body width, and body weight (Figure 12). Compared with males, females were significantly greater in weight (♀ *n* = 5, $\bar{x}$ = 6.22 mg, *sd* = 0.476; ♂ *n* = 14, $\bar{x}$ = 4.61 mg, *sd* = 1.024; *t* = 3.332, *p* < 0.001), body length (♀ $\bar{x}$ = 4.934 mm, *sd* = 0.151; ♂ $\bar{x}$ = 4.304 mm, *sd* = 0.192; *t* = 6.583, *p* < 0.001), and body width (♀ $\bar{x}$ = 4.076 mm, *sd* = 0.128; ♂ $\bar{x}$ = 3.713 mm, *sd* = 0.220; *t* = 3.441, *p* < 0.001), with all differences reaching significant levels, indicating a pattern of female-biased sexual size dimorphism. In contrast, the body length–width ratio did not differ between sexes (♀ $\bar{x}$ = 1.212, *sd* = 1.162; ♂ $\bar{x}$ = 1.162, *sd* = 0.065; *p* = 0.164), indicating that the larger body size in females might result from isometric scaling without alterations in body shape.

*N. sauteri* also exhibited significant sexual dimorphism in terms of wing length and width, whereas no significant difference was detected in the wing length–width ratio (File S3). Compared with male individuals, females presented significantly greater values of both wing length (♀ $\bar{x}$ = 3.373 mm, *sd* = 0.173; ♂ $\bar{x}$ = 3.006 mm, *sd* = 0.126; *t* = 5.101; *p* < 0.001) and wing width (♀ $\bar{x}$ = 2.058 mm, *sd* = 0.029; ♂ $\bar{x}$ = 1.916 mm, *sd* = 0.123; *t* = 4.010; *p* < 0.01). Furthermore, no significant difference in the wing length–width ratio (♀ $\bar{x}$ = 1.639 mm, *sd* = 0.089; ♂ $\bar{x}$ = 1.572 mm, *sd* = 0.070; *t* = 0.068; *p* = 0.102) was detected between the sexes.

### 3.2. Validation of Rensch’s Rule in Cassidinae Species

**Figure 13:**
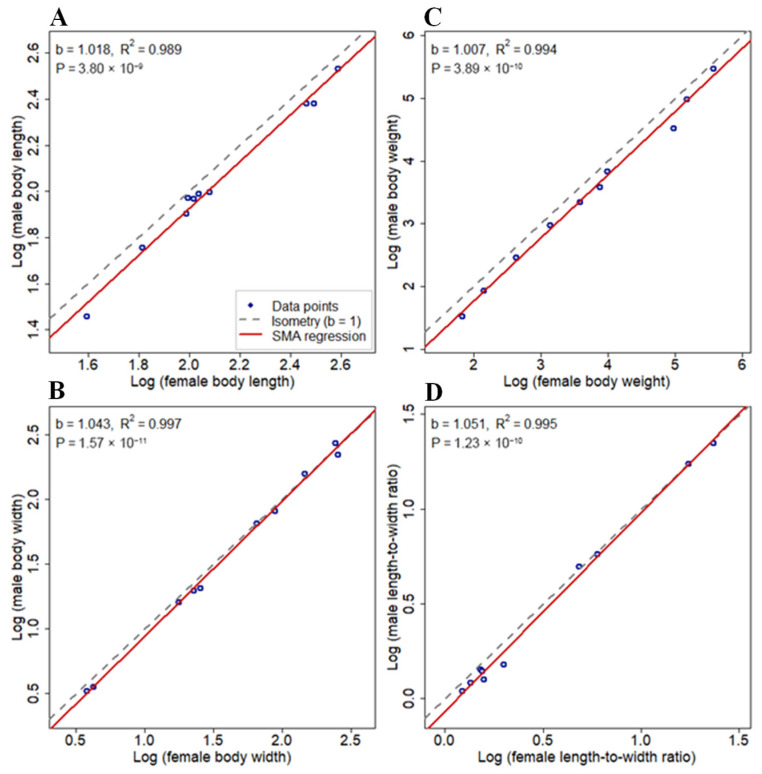
Allometric scaling of sexual size dimorphism in Cassidinae beetles. Standardized major axis regressions between log-transformed female and male body dimensions across ten species: (**A**), body length; (**B**), body width; (**C**), body weight; (**D**), length–width ratio. The solid line indicates the SMA fit; the dashed line shows isometry (slope = 1).

The results indicated that the estimated SMA slopes (*b*) for all the body size parameters were greater than 1 (Figure 13), with statistically significant overall regression fits (all *p* values for overall model fit < 0.001, *R*^2^ > 0.989). However, when testing the hypothesis that each slope equals 1 (i.e., isometry), none of the parameters significantly deviate from isometry (all *p* values for slope = 1 > 0.05). Specifically, the *p* values for the slope tests were 0.649 for body length, 0.054 for body width, 0.812 for body weight, and 0.073 for the length-to-width ratio. Thus, the male–female scaling relationships did not exhibit significant allometry in any of the four metrics, indicating a lack of directional scaling consistent with Rensch’s rule.

In contrast, the SMA slope (*b*) for all three wing parameters is less than 1, with all overall models fitting *p* < 0.001 and *R*^2^ > 0.994 (File S4). However, none of the wing parameters significantly deviated from isometry, leading to the same overall conclusion as for body size.

## 4. Discussion

Our study revealed a consistent pattern of female-biased sexual size dimorphism (SSD) across ten Cassidinae species from the eastern Nanling Mountains. Relative sex size differences indicate that females are larger than males for most morphological traits (File S5). These findings align with previous reports suggesting that Cassidinae females are typically 10–20% longer than males [54]. The body length-to-width ratio also generally reflects a more elongated shape in females. Nevertheless, male and female size distributions show substantial overlap across many species (Figure 1, Figure 2, Figure 3, Figure 4, Figure 5, Figure 6, Figure 7, Figure 8, Figure 9 and Figure 10, File S3). Given the inherently small body size of most Cassidinae beetles, visual sex identification on the basis of external morphology alone is virtually impossible [28]. The general absence of enlarged male mandibles or other conspicuous sexually dimorphic traits in Cassidinae suggests that their sexual size dimorphism (SSD) is unlikely to be driven primarily by intrasexual combat or strong mate choice—processes typically associated with such exaggerated structures [23]. Instead, the consistent pattern of larger females, particularly the pronounced disparity in body mass, is consistent with the fecundity advantage hypothesis, which posits that a larger female body size enhances reproductive output [29,32,55,56]. While we did not measure direct fecundity traits (e.g., egg counts), this interpretation aligns with patterns observed across many phytophagous beetles, where female-biased SSD often correlates with selection for increased egg production. However, alternative mechanisms—such as sex-specific developmental trajectories, differential growth rates, or ecological niche partitioning—cannot be ruled out and may also contribute to the observed dimorphism [29,32,55]. The prevalence of female-biased SSD across multiple chrysomelid subfamilies, including Cassidinae [28,54,56,57], further underscores that divergent selective regimes, rather than sexual selection alone, likely shape body size evolution in lineages lacking overt secondary sexual traits.

Our allometric analyses revealed that, across the ten Cassidinae species examined, the scaling relationship between male and female body size was statistically indistinguishable from isometry for all seven morphometric traits (i.e., slopes did not differ significantly from 1). This pattern does not conform to the prediction of Rensch’s rule—which, in systems with female-biased sexual size dimorphism (SSD), would expect a slope less than 1 (indicating that SSD decreases with increasing body size). Although our study revealed pronounced and variable female-biased SSD in this group (File S5), we found no significant evidence that the magnitude of dimorphism scales with overall body size. This result is consistent with growing evidence that Rensch’s rule may not be universally applicable across insects [33], although we caution that our modest species-level sample size limits the power to detect subtle allometric trends. Thus, while our data do not support Rensch’s rule in this Cassidinae assemblage, they should be interpreted as indicative of size-invariant SSD rather than definitive evidence of evolutionary “deviation.”

Our study has several important limitations. First, field sampling was restricted to a single 12-month period in the eastern Nanling Mountains, which limited both species representation and sample sizes per species. This constraint is particularly relevant for species such as *Callispa dimidiatipennis*, for which only 11 specimens (7 males, 4 females) were available; such small samples may reduce the robustness of intraspecific allometric estimates and could influence conclusions regarding scaling patterns. Second, the smallest Cassidinae species (generally <4 mm) were excluded because we could not reliably determine sex—despite repeated attempts—due to the technical difficulty of dissecting and recovering intact genitalia from such minute samples. Third, our interspecific test of Rensch’s rule was based on conventional allometric regression without phylogenetic correction, as a robust, time-calibrated molecular phylogeny for the focal taxa is currently unavailable. Fourth, morphological assessment relies solely on traditional linear measurements and live fresh mass for body weight, the latter of which may reflect short-term physiological variation (e.g., hydration or gut content). That said, the consistent direction and magnitude of sexual dimorphism across traits—and its concordance with structurally stable linear dimensions—suggests that our core pattern of female-biased SSD is unlikely to be entirely an artifact of these methodological constraints.

We view this work as a foundational step toward understanding SSD in Cassidinae. Future studies should expand geographic and temporal sampling to obtain larger sample sizes—preferably at least 25 individuals per sex for each focal species, as recommended—to improve the precision and reliability of allometric comparisons. Such efforts should also incorporate dry mass or standardized condition metrics, apply geometric morphometrics, and—critically—develop molecular phylogenies to enable phylogenetically informed analyses of allometric scaling and evolutionary drivers of dimorphism in this morphologically conservative but ecologically diverse beetle group.

## 5. Conclusions

This study provides one of the first multispecies, quantitative assessments of sexual size dimorphism (SSD) and allometric scaling in Cassidinae beetles. Across ten species from the eastern Nanling Mountains, we document a consistent yet variable pattern of female-biased SSD, with body mass exhibiting the greatest relative disparity. While this pattern is consistent with the fecundity advantage hypothesis, direct reproductive data (e.g., egg counts) are lacking, and alternative mechanisms cannot be excluded. Furthermore, the allometric relationship between male and female body size was statistically indistinguishable from isometry, providing no significant support for Rensch’s rule in this female-biased system. Despite methodological limitations—including limited species sampling, the absence of phylogenetic correction, and the use of live fresh mass—our findings offer a foundational framework for understanding how sexual dimorphism evolves in morphologically conserved herbivorous beetles. Future work integrating broader taxonomic sampling, molecular phylogenies, and standardized morphometric approaches will be essential to test these patterns more robustly.

## Figures and Tables

**Table 1:** Taxonomic and collection information for each Cassidinae species.

Species	Tribe	Collection Location
*Aspidomorpha sanctaecrucis* (Fabricius, 1792)	Aspidomorphini	Shixing, Guangdong, China
*Laccoptera quadrimaculata* (Thunberg, 1789)	Aspidomorphini	Shixing, Guangdong, China
*Basiprionota bisignata* (Boheman, 1862)	Basiprionotini	Dayu, Jiangxi, China
*Basiprionota chinensis* (Fabricius, 1798)	Basiprionotini	Dayu, Jiangxi, China
*Callispa dimidiatipennis* Baly, 1858	Callispini	Dinghu, Guangdong, China
*Thlaspida biramosa* (Boheman, 1855)	Cassidini	Shixing, Guangdong, China
*Downesia tarsata* Baly, 1869	Gonophorini	Chongyi, Jiangxi, China
*Monohispa tuberculata* (Gressitt, 1950)	Hispini	Quannan, Jiangxi, China
*Leptispa longipennis* (Gestro, 1890)	Leptispini	Qingxin, Guangdong, China
*Notosacantha sauteri* (Spaeth, 1914)	Notosacanthini	Nanxiong, Guangdong, China

## Data Availability

The original contributions presented in this study are included in the article/Supplementary Materials. Further inquiries can be directed to the corresponding author.

## Supplementary Materials

The following supporting information can be downloaded at https://www.mdpi.com/article/10.3390/insects17020208/s1; File S1. Body size of Cassidinae beetles. File S2. Wing size of Cassidinae beetles. File S3. Wing morphological dimorphism between sexes in different Cassidinae beetles. File S4. Allometric scaling of wing sexual size dimorphism in Cassidinae beetles. File S5. Relative sex size differences in Cassidinae beetles.
