# Supplementary material for: Beyond Rensch’s Rule: Prevalent Female-Biased Size Dimorphism and Its Allometric Scaling in Cassidinae Beetles
Source: Insects. 2026 Feb 16;17(2):208. doi: 10.3390/insects17020208 (PMC12940685; doi:10.3390/insects17020208)
Supplement: Supplementary file 1 [file insects-17-00208-s001.zip › File S3 Wing morphological dimorphism between sexes in different Cassidinae beetles.pdf]

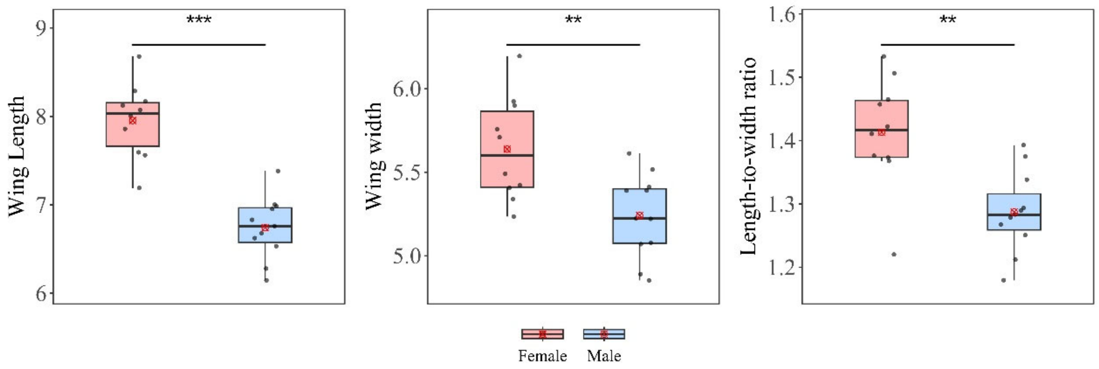


Figure S1. Wing morphological dimorphism between sexes in *Aspidomorpha sanctaecrucis*. The chart formats, statistical methods, and significance symbols are shown in Fig. 3.

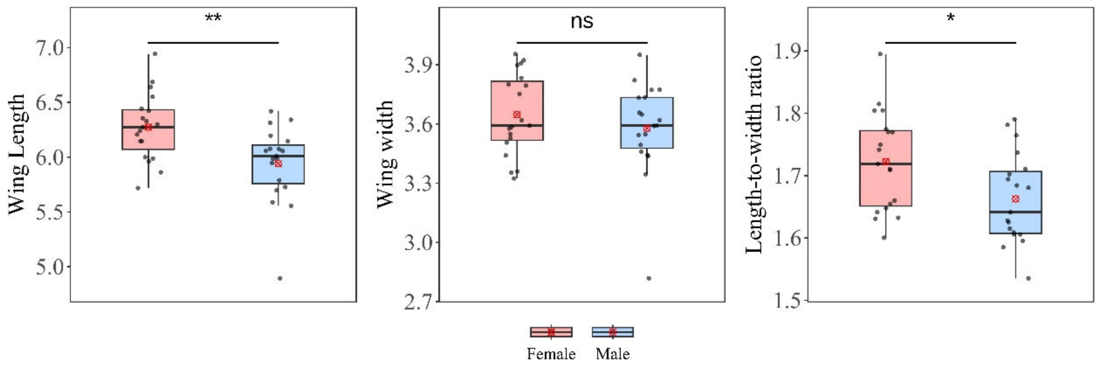


Figure S2. Wing morphological dimorphism between sexes in *Laccoptera quadrimaculata*. The chart formats, statistical methods, and significance symbols are shown in Fig. 3.

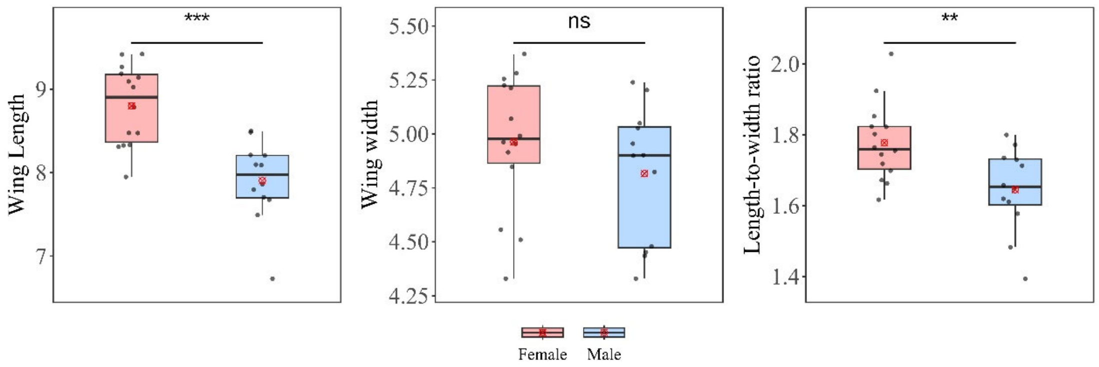


Figure S3. Wing morphological dimorphism between sexes in *Basiprionota bisignata*. The chart formats, statistical methods, and significance symbols are shown in Fig. 3.

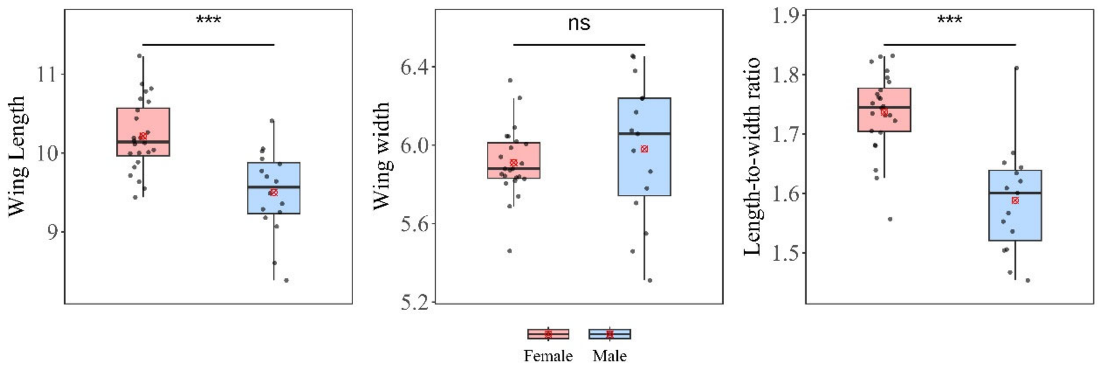


Figure S4. Wing morphological dimorphism between sexes in *Basiprionota chinensis*. The chart formats, statistical methods, and significance symbols are shown in Fig. 3.

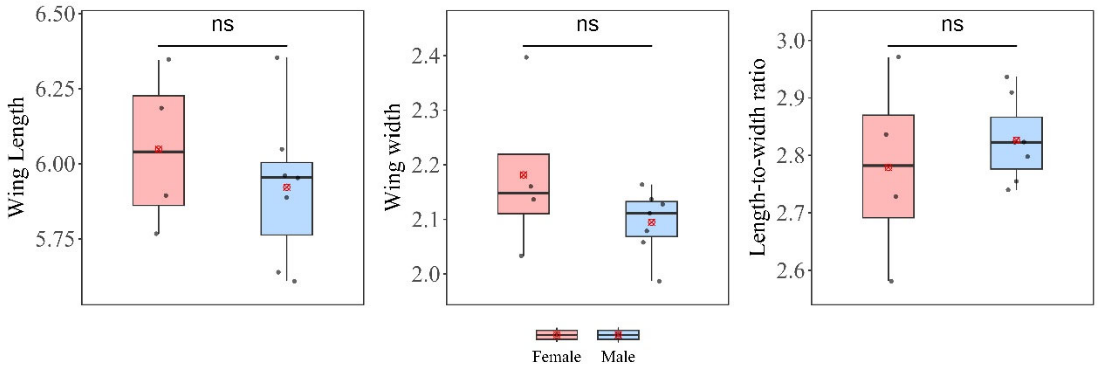


Figure S5. Wing morphological dimorphism between sexes in *Callispa dimidiatipennis*. The chart formats, statistical methods, and significance symbols are shown in Fig. 3.

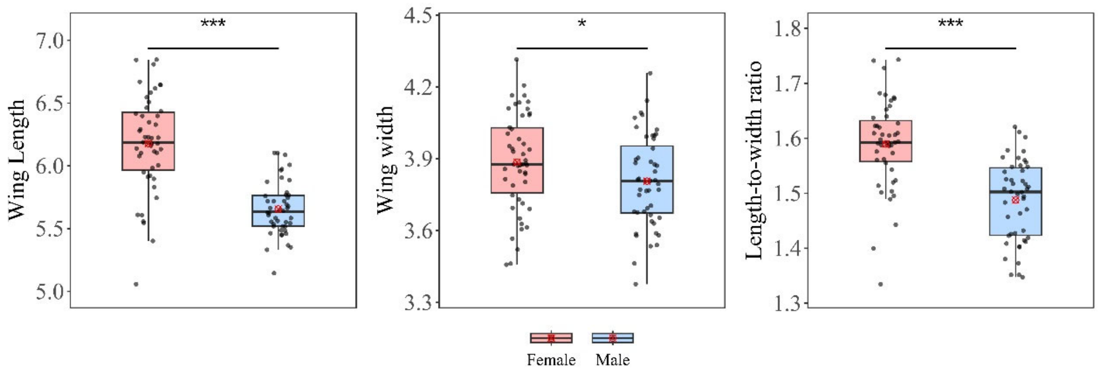


Figure S6. Wing morphological dimorphism between sexes in *Thlaspida biramosa*. The chart formats, statistical methods, and significance symbols are shown in Fig. 3.

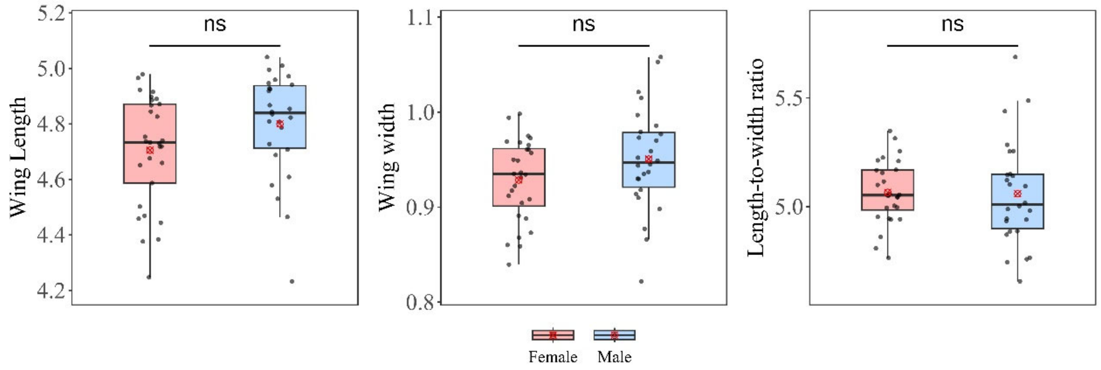


Figure S7. Wing morphological dimorphism between sexes in *Downesia tarsata*. The chart formats, statistical methods, and significance symbols are shown in Fig. 3.

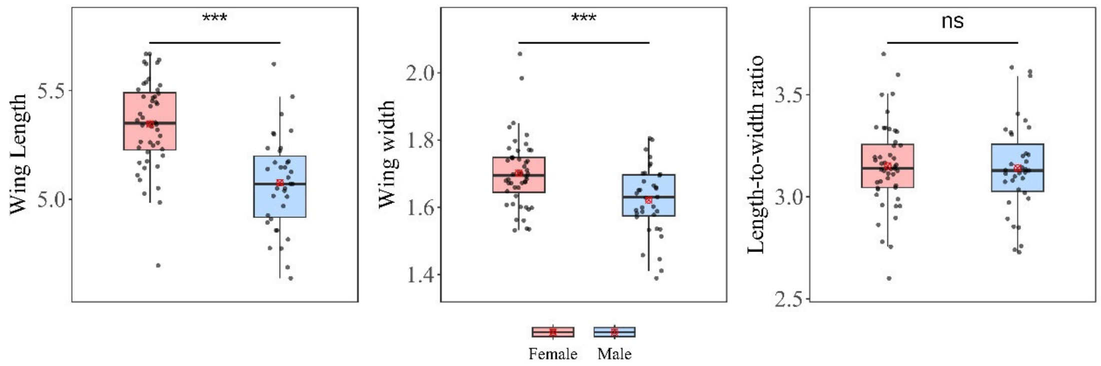


Figure S8. Wing morphological dimorphism between sexes in *Monohispa tuberculata*. The chart formats, statistical methods, and significance symbols are shown in Fig. 3.

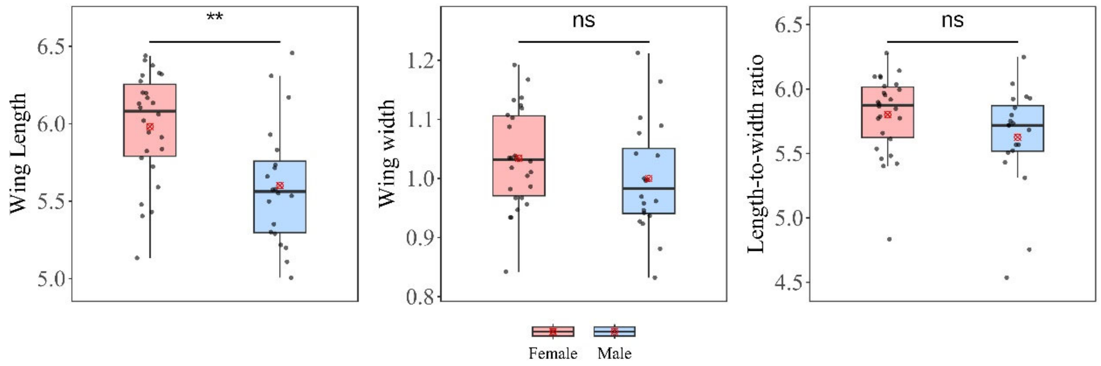


Figure S9. Wing morphological dimorphism between sexes in *Leptispa longipennis*. The chart formats, statistical methods, and significance symbols are shown in Fig. 3.

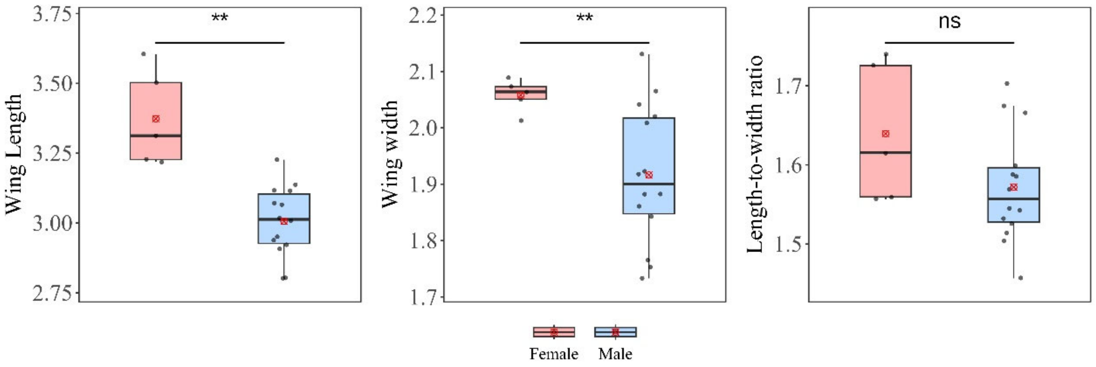


Figure S10. Wing morphological dimorphism between sexes in *Notosacantha sauteri*. The chart formats, statistical methods, and significance symbols are shown in Fig. 3.
