# Supplementary figures and images for: Beyond Rensch’s Rule: Prevalent Female-Biased Size Dimorphism and Its Allometric Scaling in Cassidinae Beetles
Source: Insects. 2026 Feb 16;17(2):208. doi: 10.3390/insects17020208 (PMC12940685; doi:10.3390/insects17020208)

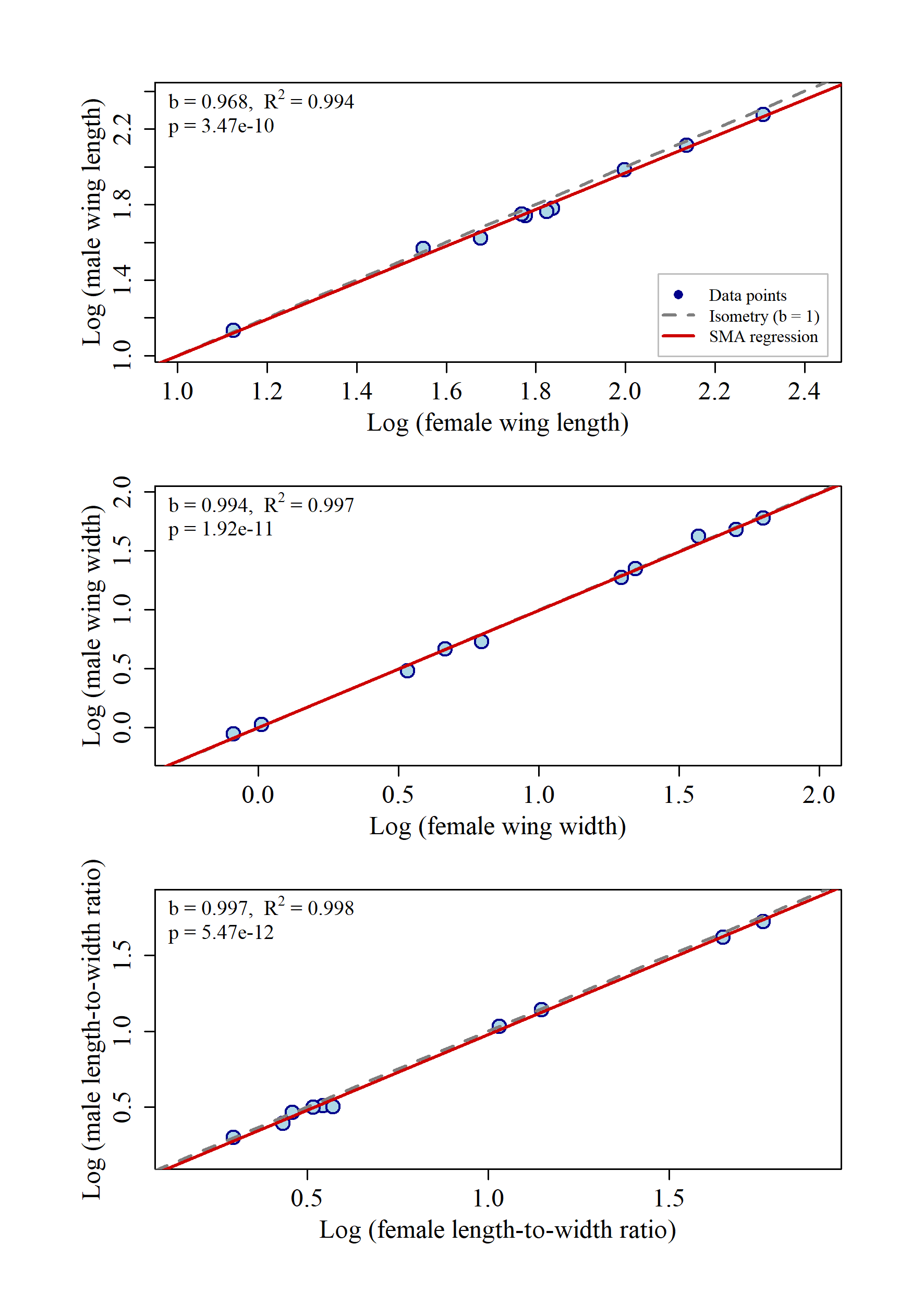

Supplement: Supplementary file 1 [file insects-17-00208-s001.zip › File S4 Allometric scaling of wing sexual size dimorphism in Cassidinae beetles.png]
